# A Comprehensive Computational Screening of Phytochemicals Derived from Saudi Medicinal Plants against Human CC Chemokine Receptor 7 to Identify Potential Anti-Cancer Therapeutics

**DOI:** 10.3390/molecules26216354

**Published:** 2021-10-21

**Authors:** Faris Alrumaihi

**Affiliations:** Department of Medical Laboratories, College of Applied Medical Sciences, Qassim University, Buraydah 51452, Saudi Arabia; f_alrumaihi@qu.edu.sa

**Keywords:** CC chemokine receptor 7, cancer, Saudi medicinal plants, natural products, virtual screening, MD simulations

## Abstract

Homeostatic trafficking of immune cells by CC chemokine receptor 7 (CCR7) keeps immune responses and tolerance in a balance. The involvement of this protein in lymph node metastasis in cancer marks CCR7 as a penitential drug target. Using the crystal structure of CCR7, herein, a comprehensive virtual screening study is presented to filter novel strong CCR7 binding phytochemicals from Saudi medicinal plants that have a higher binding affinity for the intracellular allosteric binding pocket. By doing so, three small natural molecules named as Hit-1 (1,8,10-trihydroxy-3-methoxy-6-methylanthracen-9(4H)-one), Hit-2 (4-(3,4-dimethoxybenzyl)-3-(4-hydroxy-3-methoxybenzyl)dihydrofuran-2(3H)-one), and Hit-3 (10-methyl-12,13-dihydro-[1,2]dioxolo[3,4,5-de]furo[3,2-g]isochromeno[4,3-b]chromen-8-ol) are predicted showing strong binding potential for the CC chemokine receptor 7 allosteric pocket. During molecular dynamics simulations, the compounds were observed in the formation of several chemical bonding of short bond distances. Additionally, the molecules remained in strong contact with the active pocket residues and experienced small conformation changes that seemed to be mediated by the CCR7 loops to properly engage the ligands. Two types of binding energy methods (MM/GBPBSA and WaterSwap) were additionally applied to further validate docking and simulation findings. Both analyses complement the good affinity of compounds for CCR7, the electrostatic and van der Waals energies being the most dominant in intermolecular interactions. The active pocket residue’s role in compounds binding was further evaluated via alanine scanning, which highlighted their importance in natural compounds binding. Additionally, the compounds fulfilled all drug-like rules: Lipinski, Ghose, Veber, Egan, and Muegge passed many safety parameters, making them excellent anti-cancer candidates for experimental testing.

## 1. Introduction

The human immune system has the potential to fight against pathogens without harming normal cells and tissues. One of the most important aspects is chemotactic trafficking, which guides immune cells to perform their action at appropriate places and times [1]. Chemotactic trafficking is regulated by 20 G protein-coupled receptors (GPCRs) and more than 40 chemokines. Inflammatory chemokines and receptors are generated in response to inflammatory stimuli, whereas homeostatic chemokines are produced continuously, which direct cells to certain organs [2]. Chemokine ligands such as CCL19 and CCL21 bind the CC chemokine receptor 7 (CCR7) to direct B cells, T cells, and dendritic cells to lymph nodes all across the body. The chemotactic trafficking creates cellular pathways, wherein the inflammatory immune response is induced by inflammation chemokines and target specific cells. CCL19 and CCL21 are the cellular cation receptors that, when attached to CCR7, navigate cellular and humoral immunity along with dendritic cells toward the host lymph system [3]. 

CCR7 is expressed in various lymphoid tissues. Ligands for this receptor are CCL21 (6-Ckine, SLC, TCA4, and Exodus-2) and CCL19/ECL, CK beta-11 (Exodus-3). Upregulation of CCR7 is noticed in three different types of culture: i) mouse epidermal Langerhans cells; (ii) human monocyte-derived dendritic cells; and (iii) mouse bone marrow-derived dendritic cells [4]. CCR7 and its specific ligands are important in rheumatoid arthritis (RA), which is an autoimmune disorder. Furthermore, disease-causing bacteria can use CCR7-mediated relocation of dendritic cells to drain lymph nodes, from where they move to other organs [3,5]. CCR7 is also implicated in different cancers [6,7,8,9], where chemotactic trafficking allows cancer cells to spread [10,11]. Expression of CCR7 in colon cancer is also studied [7,12]. Targeting CCR7 by low molecular weight compounds is proposed and, by doing so, may play a major role in reducing the spread of cancer cells to lymph nodes, which is an important reason for cancer related deaths [1]. Other chemokine receptors, such as CXCR1, CXCR2, CCR5, and CXCR4, are also reported to have an association with cancer spread [13]. 

The crystal structure of CCR7 is available in the protein data bank [14] and could serve as an excellent starting receptor to be used in computational drug design methods. In advanced drug development strategies, structural-based drug design is an effective way to accelerate lead molecule identification [15,16,17]. Considering this, in this study, we performed structurebased virtual screening of phytochemicals collected from Saudi medicinal plants [18]. These phytochemicals are not often explored and have a wide range of pharmaceutical applications. The shortlisted Hits, which showed a greater binding affinity for the allosteric binding pocket of CCR7, were complexed to the receptor molecule and subjected to dynamics understanding through molecular dynamics simulation. Additionally, extensive binding of free energies was performed to validate docking and simulation findings. In the end, pharmacokinetics and medicinal properties of the compounds were disclosed to guide experimentations for in vivo and in vitro studies.

## 2. Results and Discussion

### 2.1. Library Filtration

Filtration of the in-house generated drug library was first performed to remove non-drug-like molecules. This step holds great importance, as drug-like molecules enhance the chances to forward only those molecules that could lead to successful drug discovery. Out of 1741, a total of 1080 molecules were found to completely follow the Lipinski rule of five [19]. Only drug-like molecules were selected and used in the virtual screening process.

### 2.2. Molecular Docking Analysis

Target-based virtual screening was performed to identify the best binding from the Saudi medicinal plant library. The filtered compounds from the library done in the previous step were used only in the virtual screening process. The first three Hits that were ranked as best binding molecules considering their lower binding energy value were selected for binding mode and interactions analysis (Table 1).

**Figure 1:**
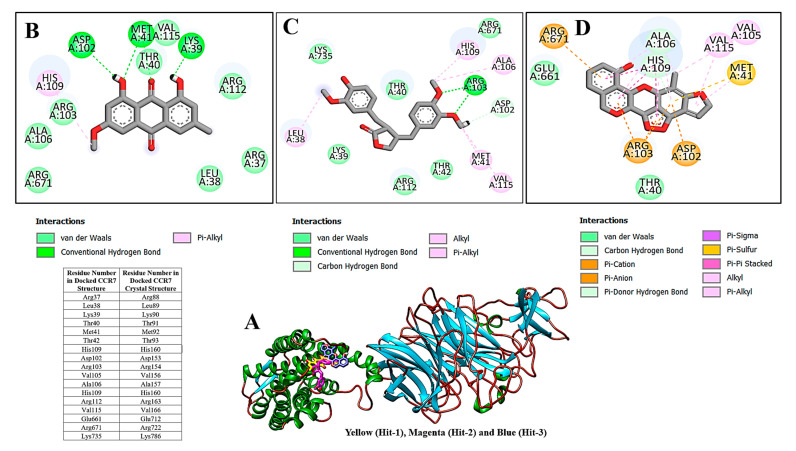
(**A**) Binding of compounds with the enzyme. Chemical interactions of Hit-1 (**B**), Hit-2 (**C**), and Hit-3 (**D**) with enzyme active site residues. Table mentioning residues numbering in the docked CCR7 structure and crystal CCR7 structure is also provided.

All three compounds bound deep inside the binding pocket near the TM6 IP linker (Figure 1A). The first Hit molecule is 1,8,10-trihydroxy-3-methoxy-6-methylanthracen-9(4H)-one, where major contribution was noticed from the central oxygen atoms and hydroxyl groups of the benzene rings (Figure 1B). The compound is strongly engaged by three hydrogen bonds of residues (residue number in crystal CCR7); Lys39 (Lys90), Met41 (Met92), and Asp102 (Asp153), with bond distances of 2.1 A, 1.8 A, and 1.9 A, respectively. Besides hydrogen bonding, several van der Waals bondings were witnessed, for example, Arg37 (Arg88), Leu38 (Leu89), Thr40 (Thr91), Arg103 (Arg154), Ala106 (Ala157), His109 (His160), Arg112 (Arg163), and Arg671 (Arg722). The Hit-2 molecule is 4-(3,4-dimethoxybenzyl)-3-(4-hydroxy-3-methoxybenzyl)dihydrofuran-2(3H)-one. After thorough search in PubChem, it has been found that it is Arctigenin, a potent ingredient of *Arctium lappa*. This compound is reported to show anti-inflammatory activity by blocking the function of ROS-dependent signal transducers and activators of transcription. It is also reported for antibacterial, antifungal, anti-cancer, and anti-diabetic activities [20,21]. The PubChem ID for this compound is 230232. The molecule was observed to dock at the same position as Hit-1; however, due to the difference in the binding conformation, the binding interaction pattern of Hit-2 is slightly different (Figure 1C). The 1,2-dimethoxy-4-methylbenzene moiety of the compound was seen in maximum interactions with the enzyme. One strong hydrogen bond interaction of the compound with Arg103 (Arg154) was seen. The rest of the compound structure (3-(4-hydroxy-3-methoxybenzyl)dihydrofuran-2(3H)-one) is engaged by many other van der Waals and very weak alky and pi-alkyl bonding. The third molecule is 10-methyl-12,13-dihydro-[1,2]dioxolo[3,4,5-de]furo[3,2-g]isochromeno[4,3-b]chromen-8-ol. The interactions of this molecule with the enzyme active pocket are dominated by very weak van der Waals, carbon hydrogen, pi-cation, pi-anion, pi-sigma, pi-sulfur, pi–pi stacked, alkyl, and pi-alkyl interactions (Figure 1D). The co-crystalized Cmp2105 compound with CCR7 outcompetes the CCL19 (native protein ligand) and has IC_50_ value of 35 nM in membrane competition experiments. Chemically, Cmp2105 contains thiadiazole-dioxide core motif along with two amine-linked substituents. The CCR7-Cmp2105 crystal structure demonstrated that the compound is bound to the same intracellular part as found for the compounds screened in this study. The binding pocket of the compounds is located between TM1, TM2, TM3, and TM6 end and between the H8 and TM7 loop of the CCR7. The Cmp2105 and all three Hits were reported to interact with the same active pocket residues. The Cmp2105 interacts with Val79, Thr82, Phe86, Thr91, Thr93, Arg154, and Tyr326. All the screened Hits as well as the Cmp2105 interact with highly conserved TM7-H8 patch residues [1]. The mentioned patch is located within the allosteric binding pocket and represents a promising hotspot for targeting chemokine receptors. Thus, it can be speculated that the filtered compounds might serve as broad spectrum blockers of CCR. In short, it can be concluded that the compounds showed favorable interactions with the enzyme active site residues and showed good binding affinity. Hit-1 and Hit-3 seem to be novel, as no such structures were found after a thorough search against PubChem and ChEMBL databases.

### 2.3. Molecular Dynamics Simulation

**Figure 2:**
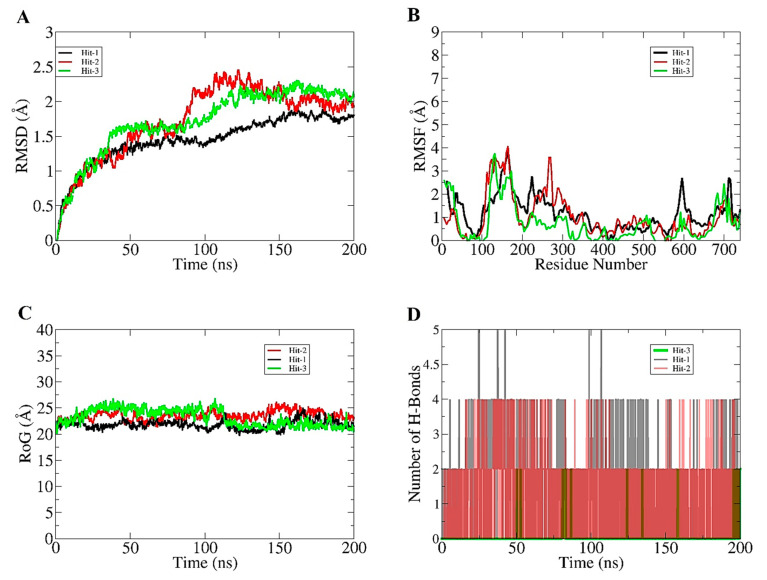
Molecular dynamics simulation analysis of selected three Hits with CCR7. (**A**) RMSD, (**B**) RMSF, (**C**) RoG, and (**D**) hydrogen bond analysis.

To examine the stability and dynamics of CCR7-compound complexes, 200 ns of molecular simulation was performed. Different statistical analysis, including root mean square RMSD [22], root mean square fluctuation (RMSF) [23], radius of gyration (RoG) [24], and hydrogen bond analysis, were performed on the simulation trajectories to decipher intermolecular strength of interaction and stable dynamics of complexes. The lower the RMSD, more stable the CCR7 will be in the presence of compounds, and vice versa. The RMSD of all three complexes was observed to be stable until 75 ns and then behaved differently for each complex and stayed constant after 150 ns till the simulation end time with no substantial variation. Between the period of 75 ns to 150 ns, the complexes experienced some deviations that were due to the protein loops, which allowed better accommodation of the compounds at the docked site. The net RMSD of Hit-1, Hit-2, and Hit-3 is 0.9 A, 1.3 A, and 1.26 A, respectively. The RMSD plot for the systems can be visualized in Figure 2A. Next, RMSF for the complexes was calculated to determine residue specific deviation in the presence of compound at the active pocket of the enzyme. As can be seen in Figure 2B, the majority of the receptor residues are in good stable range, with RMSF < 2 A. Some residues, mainly those that are part of the CCR7 loops, drive some deviations responsible for higher RMSF. The net RMSD of Hit-1, Hit-2, and Hit-3 is 0.8 A, 1.1 A, and 1.3 A, respectively. The CCR7 compactness during simulation time was then calculated through RoG. Higher RoG values indicate a lower compactness of receptor molecule, whereas lower RoG implies a higher compact nature of CCR7. As can be noticed in Figure 2C, all three systems revealed a very stable RoG plot, which is a strong indication of receptor equilibrium in the presence of compounds during simulation time. These results also complement the RMSD analysis, which depicted the stable behavior of the systems. The mean RoG values of the systems are: Hit (22.5 A), Hit-2 (23.4 A), and Hit-3 (24.42 A). The intermolecular interaction strength was determined through hydrogen bond analysis. Hydrogen bonds are key interactions that keep the docked ligands intact at the docked site and play a significant role in long term receptor-ligand stability. All three complexes reported multiple hydrogen bonds during simulation (Figure 2D). This shows that hydrogen bonding is significant in making the compounds strongly attached to the CCR7 active pocket residues.

### 2.4. Radial Distribution Plotting

**Figure 3:**
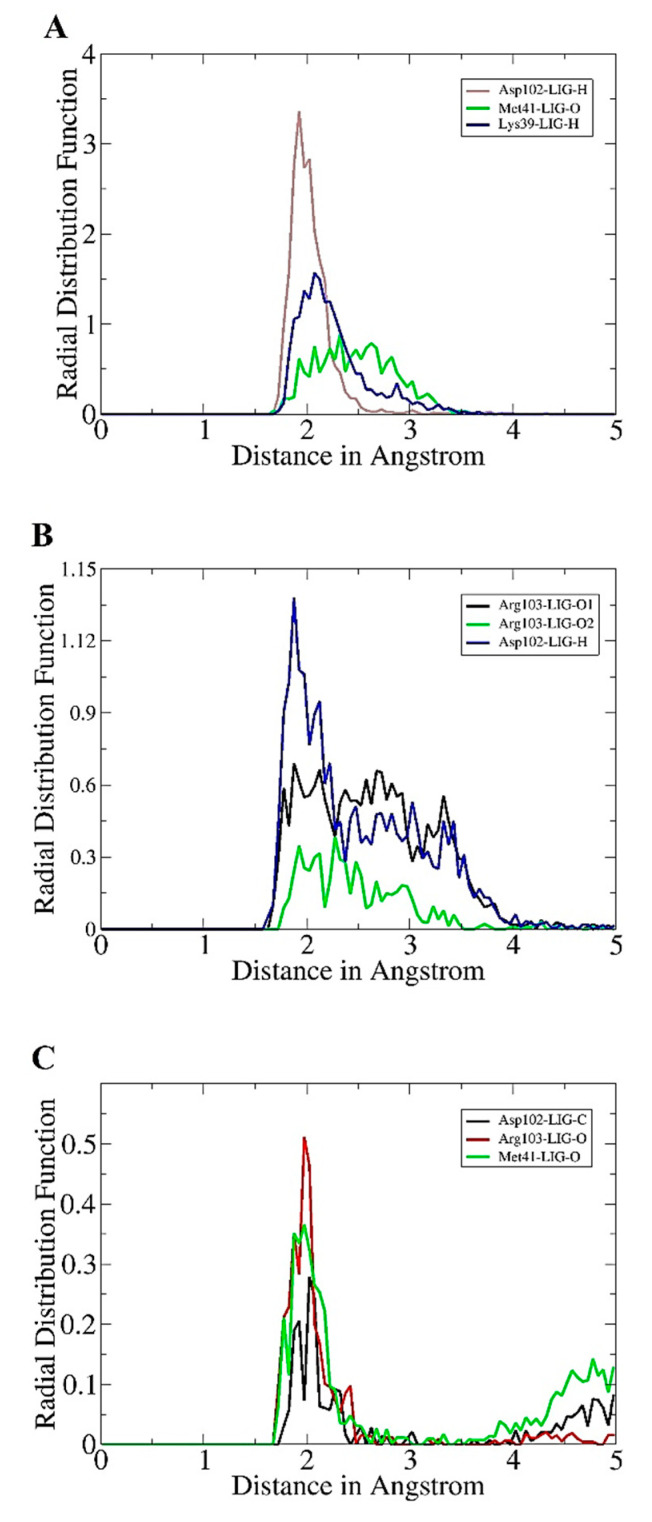
Radial distribution function analysis. (**A**) Hit-1, (**B**) Hit-2, and (**C**) Hit-3.

The radial distribution function analysis was further performed to highlight intermolecular bonds that keep the compounds in contact with enzyme active pocket residues. The intermolecular interactions were determined through an in-house script that filtered all the enzyme residues present within 3 A at the compounds bounded site. Only interactions that have high density during simulation time were used in radial distribution plotting. As can be seen in Figure 3A, for Hit-1, three interactions were reported to show high radii density. All three interactions are hydrogen bonds and are also predicted by docking and simulation studies to play role in CCR7-Hit-1 stable docking. A higher interaction density was seen between Asp102 (Asp153) and Hit-1 hydrogen atom with g(r) value > 3 at a bond distance length of ~2 A. The Met41 (Met92) and Hit-O atom interaction has a g(r) value of 1.5 and was at 2.1 A. Similarity, for Lys39 and Hit-1, hydrogen atom maximum density plotted is 0.8 at a distance of 2.3 A. In the case of the Hit-2 molecule, Asp102 (Asp153) interaction with compound hydrogen is reported to reveal high density distribution (>1.12) at a distance of 1.9 A (Figure 3B). For Hit-3, the intermolecular interaction between Asp102 (Asp153) and Hit-3 hydrogen shows a maximum g(r) value at 2 A (Figure 3C).

### 2.5. MM/GBPBSA Analysis

Both MM/GBSA and MM/PBSA methods are frequently applied in drug design works to examine drug binding affinity towards a given biological macromolecule [25]. In particular, the benefit of the methods is the use of modest computational power compared to alchemical binding free energy methods. Additionally, the methods are end point methods to test the different binding energies produced by the interaction of the compounds with the receptor. All three virtually screened compounds reported robust electrostatic and van der Waals energy; in particular, the former contributes more in the case of Hit-1, while the latter contributes significantly for Hit-2 and Hit-3. The different energy values of the compounds can be seen in Table 2. The electrostatic and van der Waal energy combine to make the gas phase energy component of the MM/GBPBSA. The net gas phase energy of Hit-1, Hit-2, and Hit-3 is –56.25 kcal/mol, −56.49 kcal/mol, and 22.39 kcal/mol, respectively. The solvation part of MM/GBPBSA was revealed to contribute negatively to overall binding energy of the complexes. In the case of MM/GBSA, the net solvation energy of Hit-1 is 14.89 kcal/mol, Hit-2 is 11.58 kcal/mol, and Hit-3 is 13.45 kcal/mol, while in MM/PBSA, Hit-1 net solvation energy is 15 kcal/mol, Hit-2 is 12.83 kcal/mol, and Hit-3 is 9.97 kcal/mol. The analysis indicated that all three compounds achieved a stable mode inside the pocket of the enzyme and are engaged by a strong chemical bonding network. However, further structure modification of these compounds can show an increased interaction pattern, which might increase their affinity towards the CCC7 receptor.

### 2.6. Enzyme Hotspot Residues

The binding energy of MMGBSA was further decomposed to dissect the energy contribution of enzyme active pocket in recognition and stabilizing compounds [26]. The binding energy of each active pocket residue in MMGBSA is tabulated in Table 3. The findings are in line with the docking predictions that highlighted several residues in compounds binding. In particular, Lys39, Met41 (Met92), Asp102 (Asp153), Arg103 (Arg154), and Arg112 (Arg163) are reported to show highly stable energy in case of Hit-1. In the case of Hit-2, Lys39 (Lys90), Thr40 (Thr91), Thr42 (Thr93), Asp102 (Asp153), Arg103 (Arg154), Arg671 (Arg722), and Lys735 (Lys786) are high-energy contributing residues. The Hit-3 molecule is strongly engaged by Lys39 (Lys90), Thr40 (Thr91), Met41 (Met92), His109 (His160), Arg103 (Arg154), Glu661 (Glu712), and Arg671 (Arg722).

### 2.7. WaterSwap Analysis

**Figure 4:**
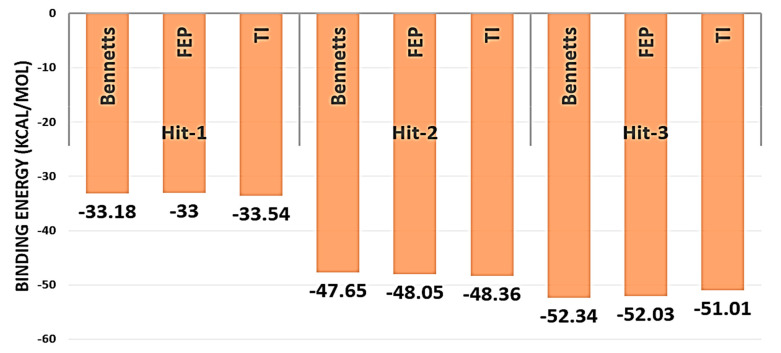
WaterSwap based estimation of binding free energy for shortlisted Hit molecules. The energy values are given in kcal/mol.

The MM/GBPBSA method, which uses an implicit water system, picked snapshots at regular intervals of molecular dynamics simulations and did not take into account the enzyme–water and drug–water interaction details. On the other hand, WaterSwap employed an explicit water model and circumvented the limitations of MM/GBPBSA [27]. Overall, the net binding free energy value for all the three systems was recorded to be <−33 kcal/mol (Figure 4). This signifies the formation of strong intermolecular affinity of the interacting enzyme and drug molecules. Additionally, it can be identified that the difference between the energy values for each complex is <1 kcal/mol, which describes good convergence of the systems.

### 2.8. Entropy Calculations

Entropy energy contribution to the systems net energy was determined through AMBER normal mode method, which predicted the net binding energy as: Hit-1 (−9.678 kcal/mol), Hit-2 (−6.894 kcal/mol), and Hit-3 (−2.3697 kcal/mol). These values suggested that, upon compounds binding, the targeted macromolecule shift the system to stable microstate level and efficient binding of the compounds to enzyme during simulation.

### 2.9. Compounds Pharmacokinetics Predictions

Computational predictions of compound pharmacokinetics holds significant importance in drug designing process, as the selection of compounds that result in poor clinical outcomes could lead to high economic lost [28,29]. Detailed pharmacokinetics of the compounds are tabulated in Table 4. All three compounds are predicted to be good drug-like molecules and follow eminent drug rules, such as Lipinski, Ghose, Veber, Egan, and Muegge [30]. All these rules determine the success of compounds in pre-clinical investigation and clinical trials and ensure the drug molecule reaches the market successfully. The compounds also showed good water solubility, which allows maximum drug concentration availability at the target site. Additionally, the compounds have high gastrointestinal absorption. Further, the compounds have zero alerts for pan-assay interference compounds (PAINS) that limited their cross interaction with non-specific biological targets and minimize the chances of false positive results. The topological surface area (TPSA) value of the compounds is within acceptable range, which affirms the compounds’ good cell membrane permeability. The compounds showed good synthetic accessibility score, which predicts that the compounds can be easily synthesized for experimental studies. From toxicity perspectives, the compounds in different toxicity tests are found to be non-toxic. They are non-hepatotoxic, non-carcinogenic in mouse, and have low *T. pyriformis* and minnow toxicity. From an excretion point of view, the compounds are good candidates to be readily excreted from the body. All the predictions indicated the compounds to be good molecules and have high chances to serve as lead molecules.

### 2.10. Alanine Scanning

Alanine scanning mutagenesis was used to get a better understanding of the contribution of active site amino acid residues to the free energy of interaction between the enzyme and compounds. The analysis suggested that a little alteration in the enzyme active pocket can affect overall binding affinity of enzyme towards ligand binding. With alanine scanning analysis, a decrease in the MMGBSA binding free energy was found for selected mutants (Lys39 (Lys90), Met41 (Met92), and Asp102 (Asp153) in the case of Hit-1; Ly39 (Lys90), Thr40 (Thr91), Thr42 (Thr93), and Arg103 (Arg154) in case of Hit-2; and Arg103 (Arg154) and Glu661 (Glu712) in case of Hit-3. The findings are congruent with the findings of the per residue energy decomposition. The substitution of alanine for active site amino acid residues resulted in a decrease in binding affinity for each active residue.

## 3. Materials and Methods

### 3.1. Phytochemicals Library Preparation

In total, a library of 1741 compounds was created by collecting phytochemicals from 63 Saudi based medicinal plants. From chemical diversity point of view, the compounds belong to alkaloids, anthraquinones, coumarins, terpenes, flavonoids, phenolic, steroids, saponins, cardenolides, tannins, phenanthrenes, glucosinolates, phenylpropanoids, polyketides, limonoids, etc. The phytochemicals database is in a process of development and will be made available soon on the web. The library was transported to LigandScout4.4 [31], where the library was filtered based on the Lipinski rule of five [19] to filter out non-drug-like molecules. The remaining molecules were then transferred to PyRx 0.8 software [32], where they were energy minimized and converted to pdbqt format.

### 3.2. Structure Based Virtual Screening

Structure-based virtual screening of filtered drug-like molecules was performed against the allosteric binding pocket of CCR7. The CCR7 crystal structure was retrieved from protein data bank in UCSF Chimera version 1.15 using PDB id of 6qzh [1]. The structure was first subjected to a short preparatory phase, where co-associated ligands were removed except for some water molecules at the active pocket, which are relevant from a function perspective. The structure was then energy minimized via steepest descent and conjugate gradient methods. The energy-minimized structure was then used in PyRx software [32]. The grid box was set at the allosteric pocket with dimension set at 15 A along XYZ dimensions. The screening process was then run, and each molecule was ranked based on binding energy in kcal/mol. For each molecule, 20 docked solutions were generated, and the one represented frequently was selected as the best binding mode. The top three best Hits were selected for further molecular dynamics simulation analysis.

### 3.3. Molecular Dynamics Simulation

Molecular dynamics simulations were accomplished for selected complexes to study the receptor/ligand stability and estimating binding free energy calculation based on the simulation trajectories [33]. Molecular dynamics simulations were carried out using AMBER20 [34]. The force field used for compounds is GAFF [35], while FF14SB [36] was employed for the CCR7 receptor. The systems were solvated into a TIP3P solvation box, large enough in size to cover the complexes. Energy minimization was done for 1000 steps through the steepest decent and conjugate gradient algorithms. In the heating step, temperature was gradually increased to 310 K through canonical ensemble. The systems were equilibrated for 500 ps, where periodic boundary conditions were applied at constant pressure, and Langevin thermostat is utilized [37]. The production run was accomplished for 200 ns for each system through isothermal and isobaric ensemble. The long-range electrostatic interactions are treated via the particle mesh Ewald method [38]. The SHAKE algorithm [39] was utilized to constrain hydrogen bonds. The simulation trajectories were then analyzed via the CPPTRAJ module [40] to investigate system structure deviations throughout the length of simulation time. The number of hydrogen bonds between the CCR7 and compounds was measured through the hydrogen bond plugin in visual molecular dynamics (VMD) version 1.9.3 software [41]. Further, the intermolecular key residue interactions were plotted in radial distribution function (g(r)) analysis [42].

### 3.4. MM/GBPBSA and Alanine Scanning Analysis

The different binding free energies between the CCR7 and compounds were estimated using an MM/GBPBSA method. The analysis was performed through MMPBSA.py module [43]. The number of frames used from simulation trajectories was 100. Entropy of the systems was calculated using AMBER normal mode analysis over 10 frames. Additionally, the key residues were mutated to alanine using alanine scanning to examine the importance of such residues in ligand binding and stability. The detail methodology used for performing MM/GBPBSA and alanine scanning was followed from Asama et al., 2017 [26].

### 3.5. WaterSwap Analysis

WaterSwap is a binding free energy predicting method that used Monte Carlo simulation to estimate binding free energies for default 1000 iterations [27,44]. The water molecule role is usually skipped in MM/GBPBSA, which is important in cases where water molecules act as birding molecules between protein–ligand interactions. Three algorithms were run during the analysis: free energy perturbation (FEP), thermodynamic integration (TI), and Bennett’s acceptance ratio (BAR). The difference of 1 kcal/mol among the mentioned methods reflects on a values convergence [45].

### 3.6. In Silico Pharmacokinetics, Medicinal Chemistry and Toxicity

To assess the feasibility of the compounds for experimentations and biological testing, in silico pharmacokinetics, medicinal chemistry, and toxicity were evaluated using pkCSM [46] and SWISSADME [47] servers.

## 4. Conclusions

Computer-aided drug design is gaining popularity to virtually screen small drug molecule libraries against a given biological time, thus saving time and economic losses associated with experimental drug design. In this present study, medicinal plant-derived phytochemicals were used in structure-based virtual screenings against an allosteric binding pocket of CCR7 receptor. This filtered three drug-like molecules that showed excellent binding affinity for the CCR7. Dynamically, the compounds’ binding was observed to be stable, and the interacting network involved short-distance hydrogen and van der Waals bindings. The binding energies by MM/GBPBSA indicated the gas phase energies (electrostatic and van der Waals) to dominate complex formation, which is additionally validated by the explicit WaterSwap method. The compounds were also predicted to follow druglike rules and have good pharmacokinetics, medicinal chemistry, and low toxicity. These findings suggest the compounds are worthy to be utilized in experimental studies to examine their potency against the CCR7 receptor.

## Figures and Tables

**Table 1:** Shortlisted top three compounds from virtual screening against the enzyme.

Compounds	Autodock Vina Binding Free Energy (kcal/mol)
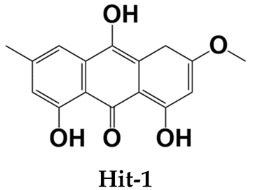	–10.97
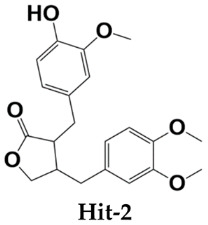	–9.71
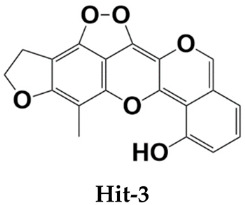	–7.00

**Table 2:** MM/GBPBSA binding free energy analysis of the compounds. Each value is shown in kcal/mol.

Compound	MM/GBSA						
	ΔG Binding	ΔG Electrostatic	ΔG Bind Van Der Waals	ΔG Bind Gas Phase	ΔG Polar Solvation	ΔG Non Polar Solvation	ΔG Solvation
Hit-1	−41.36	−30.58	−25.67	−56.25	25.17	−10.28	14.89
Hit-2	−44.91	−25.67	−30.82	−56.49	25.63	−14.05	11.58
Hit-3	−19.58	−14.58	−18.45	33.03	22.39	−8.94	13.45
	**MM/PBSA**						
Hit-1	−41.25	−30.58	−25.67	−56.25	24.64	−9.64	15
Hit-2	−43.66	−25.67	−30.82	−56.49	26.47	−13.64	12.83
Hit-3	−23.06	−14.58	−18.45	33.03	22.31	−12.34	9.97

**Table 3:** Residue wise decomposition of net MMGBSA energy into enzyme residues. Each value is presented in kcal/mol.

Ligand/Residue	Hit-1	Hit-2	Hit-3
Arg37 (Arg88)	−0.58	−0.88	0.94
Leu38 (Leu89)	−0.61	−0.71	−0.77
Lys39 (Lys90)	−3.69	−1.86	−1.00
Thr40 (Thr91)	−1.67	−2.38	−2.54
Met41 (Met92)	−4.21	1.49	−1.01
Thr42 (Thr93)	0.48	−1.63	−0.98
His109 (His160)	1.23	−0.54	−2.37
Asp102(Asp153)	−5.01	−2.74	−0.88
Arg103 (Arg154)	−2.67	−2.51	−2.35
Val105 (Val156)	0.21	−0.21	−0.62
Ala106 (Ala157)	0.42	−0.78	−0.55
Arg112 (Arg163)	−1.62	−0.54	0.24
Val115 (Val166)	0.21	0.41	0.62
Glu661 (Glu712)	1.25	−0.58	−3.24
Arg671 (Arg722)	−1.0	−1.50	−1.56
Lys735 (Lys786)	2.25	−1.36	−0.87

**Table 4:** Computational pharmacokinetics of compounds.

Property	Compounds		
Physicochemical Properties	Hit-1	Hit-2	Hit-3
Formula	C16H14O5	C21H24O6	C19H12O6
Molecular weight	286.28 g/mol	372.41 g/mol	336.29 g/mol
Num. heavy atoms	21	27	25
Num. arom. heavy atoms	6	12	18
Fraction Csp3	0.19	0.38	0.16
Num. rotatable bonds	1	7	0
Num. H-bond acceptors	5	6	6
Num. H-bond donors	3	1	1
Molar Refractivity	76.80	100.60	86.94
TPSA	86.99 A²	74.22 A²	78.11 A²
**Lipophilicity**			
Consensus Log Po/w	1.94	3.10	3.00
Water Solubility	Soluble	Moderately soluble	Soluble
**Pharmacokinetics**			
GI absorption	High	High	High
BBB permeant	No	Yes	Yes
P-gp substrate	No	No	Yes
CYP1A2 inhibitor	Yes	No	Yes
CYP2C19 inhibitor	No	No	No
CYP2C9 inhibitor	Yes	Yes	Yes
CYP2D6 inhibitor	No	Yes	No
CYP3A4 inhibitor	Yes	Yes	Yes
Log Kp (skin permeation)	–6.59 cm/s	–6.02 cm/s	–6.65 cm/s
**Drug-likeness**			
Lipinski	Yes	Yes	Yes
**Medicinal chemistry**			
PAINS	0 alert	0 alert	0 alert
Synthetic accessibility	3.50	3.43	4.12
**Toxicity**			
Hepatotoxicity	No	No	No
Skin Sensitisation	No	No	No
*T. pyriformis* toxicity	0.316 (log ug/L)	0.469 (log ug/L)	0.29 (log ug/L)
AMES toxicity	Yes	No	Yes
Minnow toxicity	1.836 (log mM)	0.482 (log mM)	–0.137 (log mM)
Carcino mouse	No	No	No
**Excretion**			
Total Clearance	0.44 (log mL/min/kg)	0.25 (log mL/min/kg)	0.093 (log mL/min/kg)
Renal OCT2 substrate	No	No	No

## Data Availability

The data presented in this study are available within the article.
